# Evaluating pharmacist independent prescribing for patients with mental illness in community care: a qualitative study

**DOI:** 10.3389/fpsyt.2025.1637132

**Published:** 2025-09-08

**Authors:** Bashayr A. Alsaeed, Jason Hall, Richard N. Keers

**Affiliations:** ^1^ Division of Pharmacy and Optometry, School of Health Sciences, Faculty of Biology, Medicine and Health, Manchester Academic Health Science Centre, The University of Manchester, Manchester, United Kingdom; ^2^ Department of Clinical Pharmacy, College of Pharmacy, Jouf University, Sakaka, Al-Jouf, Saudi Arabia; ^3^ National Institute for Health and Care Research (NIHR) Greater Manchester Patient Safety Research Collaboration, University of Manchester, Manchester, United Kingdom; ^4^ Optimising Outcomes with Medicines (OptiMed) Research Unit, Pennine Care National Health Service (NHS) Foundation Trust, Greater Manchester, United Kingdom

**Keywords:** pharmacist prescribers, pharmacist prescribing services, community mental health services, mental disorders, people with mental illness

## Abstract

**Background:**

Non-medical prescribing by pharmacists, nurses, and other professionals has been introduced over recent decades to address staff shortages and the growing demand for mental health services globally. However, most of the emerging evidence concerning the contribution and impact of non-medical prescribing focuses on nurses, despite the expanding role of pharmacists.

**Aim:**

The study aimed to explore in depth the factors influencing implementation and delivery of pharmacist non-medical prescribing services for patients with mental illness in community-based settings across the UK.

**Method:**

Remote semi-structured interviews were conducted with pharmacist independent prescribers across the UK between January and June 2024. Participants were recruited using purposive sampling through the research team’s professional networks and social media platforms, with data transcribed and analysed thematically.

**Results:**

20 pharmacist prescribers were interviewed, including six from general practice and seven from specialist mental health care. Four main themes, including insecurity, training/education, ambiguity, and workload management were identified. Lack of confidence in prescribing was reported by most participants – general practice based pharmacists cited challenges related to a lack of confidence in managing patients with mental health illness, whereas those in specialist services identified difficulties with risk management. Concerns about training and education were frequently raised by participants, including inadequacies in the undergraduate pharmacy curriculum and non-medical prescribing courses in preparing them for key elements of practice related to mental health care such as assessing patients with mental illness. Pharmacist prescribers also reported challenges with workload management and role clarity. While pharmacists anecdotally perceived high patient satisfaction with the care they provided, this was not reported to be formally evaluated.

**Conclusion:**

Several factors were identified that influenced successful implementation and delivery of pharmacist prescribing services for patients with mental illness in community care. Improved education and training in mental health along with a clearer definition of the pharmacist prescribing role may support optimal service delivery. Future work evaluating pharmacist prescribing should explore the viewpoints of patients and carers in order to develop holistic improvement recommendations driven by key stakeholders.

## Background

The demand for mental health support has increased significantly since 2019, leading to a greater need for accessible and effective mental health services (1–3). Despite high demand for mental health services, patients may experience difficulty accessing these services in a timely manner due to a shortage of medical staff, with the situation further exacerbated by the COVID-19 pandemic (4, 5). The need to ensure wider and timely access to care was a key driver for introducing non-medical prescribing, which allows trained healthcare professionals, such as pharmacists and nurses, to prescribe medications. This approach has emerged as a solution to bridge the gap in healthcare access and help address growing care demands (6).

In the United Kingdom (UK), non-medical prescribing has developed over time. Some nurses gained limited prescribing rights in the 1990s (7) before expanding in 2003 with the introduction of supplementary prescribing (8) which allowed both pharmacists and nurses to prescribe within a predefined treatment plan which had been agreed with a doctor. This was followed by independent prescribing in 2006 (9) which granted pharmacists and nurses the ability to prescribe medications autonomously within their competency. Both forms of prescribing require additional training and education from the relevant professional body (7). However, a recent change in UK pharmacy education means that, from 2026 onwards, all pharmacy graduates will qualify as independent prescribers upon registration with the General Pharmaceutical Council (GPhC). This change was designed to enhance patient care as pharmacists will be able to prescribe and manage conditions without additional postgraduate training (10). Unlike the UK which grants the most extensive non-medical prescribing rights to nurses and pharmacists, particularly through independent prescribing, prescribing rights in countries such as the United States (US), Canada, and Australia are generally more restricted and vary by region. For example, nurse practitioners in parts of the US and Canada have near-autonomous authority (11, 12), while pharmacists typically prescribe under collaborative agreements or limited conditions (13, 14). In Australia, both professions prescribe within structured arrangements with physicians (15, 16).

Since the introduction of non-medical prescribing, several studies have explored the impact of care delivered by non-medical prescribers on various patient groups. In the community, non-medical prescribing has been associated with improved access to treatment, reduced waiting times, and better information provision for patients (17–19). A recent scoping review of 63 studies reported the same positive impact of non-medical prescribing for mental health care in the community (20). However, the majority of this evidence focussed on nurse prescribers, with relatively little attention given to pharmacist prescribers. This gap is important as over the past decade, pharmacists have seen their roles supporting mental health care expand in community practice which include working as part of multidisciplinary teams, involvement in the screening and risk assessment of mental health illness, and conducting medication review services (21). A European study published in 2023 further highlights the expanding role of clinical pharmacists in mental health, for example reporting high acceptance of pharmacists’ recommendations by psychiatrists in Slovenia following medication reviews as an example of the effective involvement of pharmacists within multidisciplinary teams (22). A recent review by NHS England of role expansion for specialised mental health pharmacists highlighted examples such as providing timely access to medication and offering advice and guidance to other healthcare professionals (23). In the UK, medication reviews conducted by pharmacists may or may not include prescribing, depending on the pharmacist’s scope of practice and local regulations. Independent prescribing pharmacists can adjust or initiate medications as part of a comprehensive medication review, whereas non-prescribing pharmacists focus on optimising therapy through recommendations to the patient and/or prescriber (6). Given the increasing demand for mental health support across all age groups and the expanding role of pharmacists who may use prescribing skills for mental health care in the community, it is important to further explore their integration in this setting and the impact on this patient population.

Better understanding of the types of roles, impact and factors influencing pharmacist non-medical prescribers face when delivering care to people with mental illness in the community will guide future practice and support the development of strategies to improve their integration into the healthcare system. This in turn should support provision of timely, effective and safe services to the community care setting where patient need is greatest as approximately 90% of people with mental health conditions are managed within the community (24).

### Study aim

This study aimed to identify the factors influencing the implementation and delivery of pharmacist independent non-medical prescribing services for patients with mental illness in UK community care, and to seek insight into any impact these prescribers have on patient care.

### Study objectives

• To identify the factors influencing implementation and delivery of services for patients with mental illness provided by pharmacist non-medical prescribers in community care.
• To explore qualitatively whether pharmacist non-medical prescribing services had any impact.
• To generate recommendations to guide clinical practice, service development and research to enhance pharmacist non-medical prescribing services and optimise the delivery of care for patients with mental illness.

## Methods

### Study design and setting

This study employed a qualitative study design using semi-structured interviews. Community based settings were considered to include general practice (GP), community pharmacy, specialist community mental health services, and primary care networks (integrated and collaborative care networks). A summary of the different community-based mental health care services and primary care networks (PCNs) is provided in **Table 1**.

**Table 1:** A summary of the different types of community-based settings.

Different types of community-based care	The support they provide
Community mental health teams (CMHTs) (51)	CMHTs services focus on providing support for patient with mental illness, particularly those whose needs may not be fully met by general practice. These services also aim to promote recovery and provide ongoing, specialised care.
Crisis team (52)	Crisis teams provide intensive, short-term support to people experiencing a mental health crisis, aiming to prevent hospital admission whenever possible. However, they are also responsible for facilitating hospital admission if necessary. These teams can offer medication management, ensure coordination with other services for ongoing care, and arrange regular home visits as needed.
Early intervention team (EIT) (53)	Early Intervention Teams (EIT) consist of multidisciplinary teams, including care coordinators, psychiatrists, clinical psychologists, and other key staff, who support people experiencing a first episode of psychosis. Their aim is to provide the best available treatment, promote recovery, and prevent relapse.
Assertive outreach team (AOT) (52)	They are also called complex care teams, which focus on providing help for those who have complex mental health-related issues. They support patients with complex mental health illnesses (e.g., violent behaviour, serious self-harming, and dual diagnosis) to be able to access the services and care they need.
Primary care networks (PCNs) (54)	Primary Care Networks (PCNs) are collaborations between GP practices and other health providers typically covering populations of around 50,000 people. They aim to deliver more personalised and coordinated care by linking general practices with community services, introducing additional services, and expanding the multidisciplinary workforce at a local level.
General Practice (GP) (55)	General practice is one of the key primary care services in the UK and often serves as the first point of contact for patients. General practitioners (GPs) manage a wide range of health issues and coordinate care with other healthcare professionals when necessary, including making referrals to other services.
Community Pharmacy (56)	Community pharmacy, also known as retail pharmacy, is an integral part of the NHS primary care system. These pharmacies are highly accessible, often located on high streets, in supermarkets, and within neighbourhood centres. They provide a range of services, including essential services such as dispensing medicines and medical appliances, offering health advice, and optional advanced services such as flu vaccinations.

#### Sampling method

Participants were recruited from within the UK based on pre-defined criteria according to a purposive sampling approach, and included:

- pharmacist prescribers who cared for patients with mental illness in the community setting (when we refer to pharmacist prescribers, we are specifically meaning pharmacist independent prescribers which is now the dominant non-medical prescribing model for pharmacists in the UK), OR
- wider staff members working with these prescribers (general practitioners (GPs), nurses, community mental health team staff, or psychiatrists), OR
- managers who either worked with pharmacist prescribers or had experience introducing and/or managing their services.

Participants were sought from general practice, community pharmacy, specialist mental health community teams/outpatient services and PCNs. As recruitment progressed, due to the under representation of pharmacists working in general practices and community mental health teams, we altered our strategy to focus more on this group.

Pharmacist prescribers needed to be registered with the GPhC and have experience in caring for patients with mental illness. Recruitment took place via the professional networks of the research team, and through social media between January–June 2024. Snowballing was also utilised to reach and recruit additional participants beyond the direct network of the research team. By “research teams professionals’ networks,” we refer to national organisations with pharmacist prescribers as members such as the College of Mental Health Pharmacy (CMHP), along with promotion through colleagues. In addition, some pharmacist prescribers known to the supervisory team were approached directly. Pharmacist prescribers who had already taken part in the study were also asked to snowball the study through their own networks. We also asked colleagues to promote the study via Higher Education Institutions (HEIs), non-medical prescribing (NMP) leads in Northwest England, and the national NMP group. These efforts included national channels to reach potential participants across the UK. Interested parties were asked to read the study participant information sheet and, if willing to participate, to sign and return an electronic consent form before a remote interview was conducted using either Zoom or Microsoft Teams depending on their preference. Subsequently, the interviewer (B. Alsaeed) scheduled the remote interview at a convenient time and date for the participant.

One participant was interviewed but later excluded due to ineligibility, three were eligible and interested but did not sign the consent form, two expressed interest but were not eligible, and one signed the consent form but later dropped. In total, 20 participants were interviewed and included in the study. The interviews were conducted until data saturation was achieved. Saturation began to be observed after half of the interviews and was fully achieved by the 20th interview, when no new codes or themes emerged, aligning with previous research indicating saturation typically occurs between 9 and 17 interviews (25).

### Data collection

The interview guide was developed by the research team and can be seen in **Supplementary File 1**. One pilot interview was conducted with a mental health pharmacist to test feasibility and understanding (this was not recorded), with minor refinements to the interview guide required. Interviews began with an explanation of the study’s goals, followed by gathering background information about the participants including their care setting and roles/duties in supporting people with mental illness. This was followed by identification and exploration of any factors influencing implementation and delivery of pharmacist prescribing services provided for this patient group, as well as suggesting ways to improve the service in the future. Participants were informed, prior to and at the start of the interview, that this project formed part of the researcher’s PhD study. The interviews lasted between 17 to 61 minutes, with an average length of 37.4 minutes. All interviews were conducted remotely, as one-to-one sessions, by the main researcher (B. Alsaeed) from her home or workplace. All interviews were both audio- and video-recorded. Audio recording supported the research process by enabling the generation of precise transcripts. While video recording was valuable for observing non-verbal cues and ensuring that no one else was present with the participants. During the interviews, field notes were taken to capture additional contextual information. No repeat interviews were conducted.

### Data processing and analysis

The interview recordings were sent to a university approved transcribing service for intelligent verbatim transcription. To ensure accuracy, B. Alsaeed reviewed and checked the returned transcripts against the recordings before destroying the recordings permanently. The transcripts were not offered to participants. The interview transcripts were analysed using NVivo 12 Plus software. This study applied the six-key-steps thematic framework established for analysing qualitative data (26). These steps started with familiarisation with the data, generation of initial codes, searching for initial themes, refining of identified themes, then defining and naming the generated themes to enable writing of the study report. The themes were inductively derived from the data. The first 10 (50%) interview transcripts were independently analysed by the research team (RNK, JH) to reach consensus on the coding framework, followed by thematic analysis of the whole dataset by the main researcher (B. Alsaeed) with ongoing supervision and discussion from the research team. Throughout the analysis, the team actively sought negative and discrepant cases to challenge emerging themes and enhance the credibility of the findings. This study followed the Consolidated Criteria for Reporting Qualitative Research (COREQ) checklist (27), which is provided as **Supplementary File 2**. Participants were not involved in the data analysis process.

### Ethical consideration and approval

In this study, participants received a detailed information sheet about the study and its objectives before providing informed written consent immediately before their interview. To ensure confidentiality, interview data were pseudonymised, in which each participant was assigned a unique code that was used alongside anonymised quotations in reports and publications. A distress protocol was prepared, and participants were informed of the availability of the protocol which included the option of withdrawing from the study or continuing or rescheduling the interview later (this was not needed during the study). Ethical approval of this study was obtained from the University of Manchester research ethics committee (reference 2024-18280-33163).

### Reflexivity/positionality

The lead researcher (BA), a pharmacist qualified and registered in Saudi Arabia and a PhD candidate in Pharmacy Practice at the University of Manchester, conducted and coded all interviews. This study was conducted as part of her PhD qualification. She had formal training in qualitative research methods, including interviewing and thematic analysis, and had no prior professional or personal relationships with participants. Measures to minimise bias and enhance credibility, including independent coding and consensus processes, are described in the data processing and analysis section.

## Results

A total of 20 pharmacist prescribers were interviewed from community-based settings in the UK – 17 participants were practising in England, two were practising in Scotland, and one was practising in Northern Ireland. Of the 20 participants, six worked in general practice (including one who worked in both general practice and community pharmacy but only discussed their experience in general practice), seven were employed in community mental health teams (CMHTs), three were based in specialist perinatal community mental health teams, one worked in a crisis and psychiatric liaison community team, and three were based in PCNs. Participants varied in their years of qualification as a prescriber: three participants were qualified for 1–3 years; three for 3–5 years; ten for 5–10 years; and four for 10–20 years. Out of 20 pharmacists interviewed in this study, six had joint roles in addition to them being prescribers, these including a position in management (n=3), education (n=2), and training (n=2).

Depression and anxiety were reported by most of the participants as the most common mental illness they were managing in their practice (n=14/20 and 13/20, respectively). This was followed by bipolar disorder (n=9) and schizophrenia (n=6). Across the interviews, pharmacist prescribers were reported to be engaged in a wide range of duties. The most commonly reported were medication reviews (n=14), prescribing and initiating new medications (n=13), monitoring and follow-up (n=8), deprescribing and polypharmacy management (n=6), and clinical consultation and advice (n=6). Most pharmacists were involved in more than one of these duties.

### Factors that influenced the successful implementation and delivery of pharmacist non-medical prescribing services for patients with mental illness

**Figure 1:**
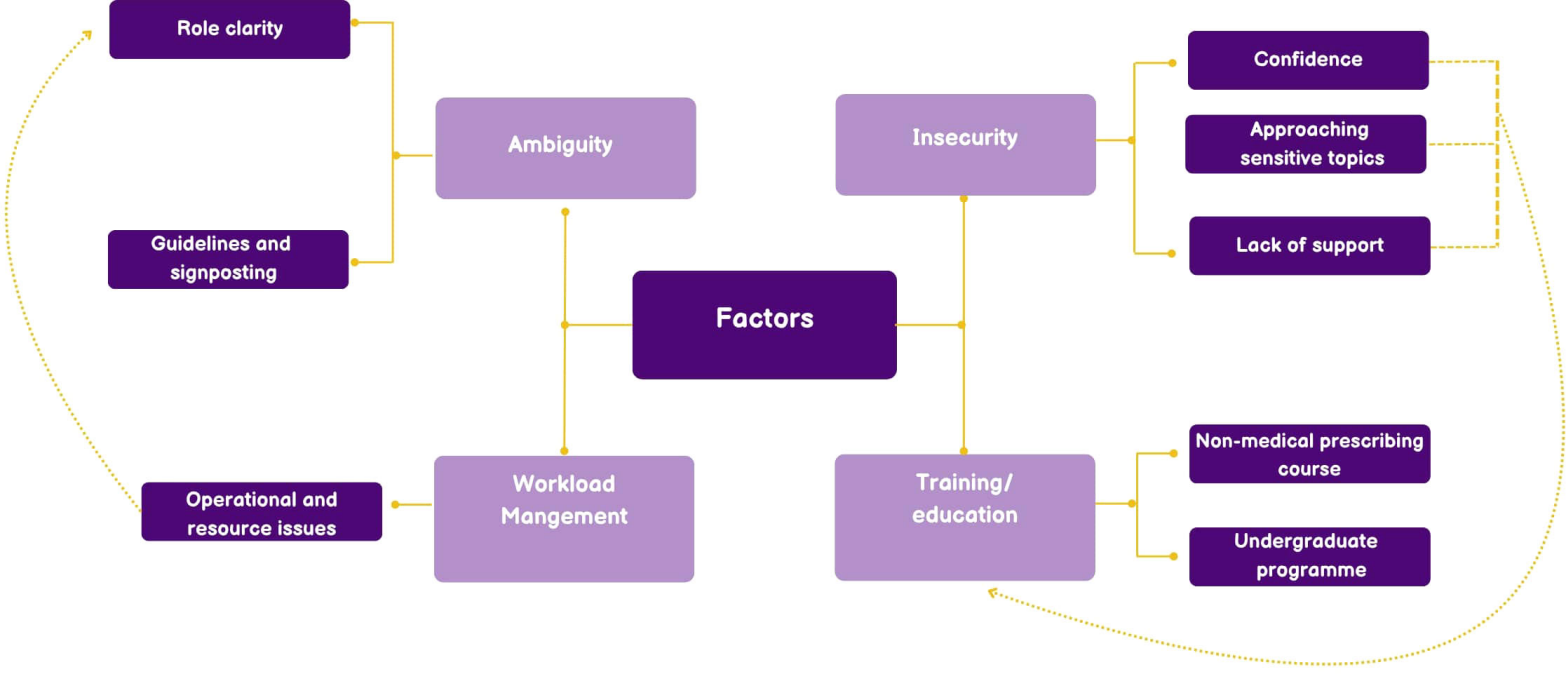
Factors influencing the successful delivery and implementation of pharmacist non-medical prescribing services for patients with mental illness.

Challenges related to implementation and delivery of their services as non-medical prescribers were discussed among all pharmacists who participated in this study. Four main themes emerged from the analysis: insecurity, ambiguity, training/education, and workload management. A full summary of the themes and subthemes is presented in **Figure 1**, with illustrative quotes provided in **Supplementary File 3**.

### Insecurity

#### Confidence

Lack of confidence was discussed by the majority of the participants (n=15/20) either at the start of their new prescribing role, or as an ongoing issue. Confidence was related to a number of factors, including the setting they worked in, the knowledge and training they had, and their exposure (experience). One general practice pharmacist who recently qualified as a prescriber reported that, despite having the ability to prescribe independently, felt concerned about making mistakes. This concern, particularly due to differences they perceived in clinical checking of prescriptions by other pharmacists between hospital and community care, left them feeling less confident to prescribe autonomously.

*“I think confidence is definitely a big one. [ … ], fear of making a mistake. Because, for example, I worked in a hospital pharmacy, and if I ever prescribed something, then another pharmacist would check my work. Whereas community pharmacists are so rushed, and they’re not particularly clinical, so I know that once I prescribe something, it’s not going to get checked, in terms of whether it’s appropriate for that specific patient. So it’s also the element of responsibility, that I don’t feel comfortable with.” [P18, General Practice]*

Patient reviews were described by a few participants as being completed remotely due to unavailability of a consultation room, which resulted in feeling less confident in prescribing, as the pharmacist was not able to see the complete picture.

*“A lot of my reviews are done over the telephone, we don’t see many patients face-to-face. [ … ]. And that’s just because of limitations. So, in this surgery, [ … ], I’m just upstairs, in an office with other admin[strative] staff. So, [ … ], not seeing patients face-to-face with them, means I’m less confident in prescribing, because I don’t quite fully know the whole picture.” [P18, General Practice]*

The vast majority of participants agreed that having adequate and consistent support and clinical supervision was crucial in becoming more confident in providing mental health care and to support development of their prescribing role. A CMHT pharmacist mentioned how clinical supervision during the start of their prescribing role from a consultant psychiatrist was helpful in growing their confidence and diagnostic skills:

*“I think after so many times of, like, just checking your plan with the consultant psychiatrist and them saying yes, I agree with what you’re doing, I would have done the same thing or yes, this is really tricky, I don’t know what to do either, over time that gave me confidence that yes, what I am doing is what another reasonable person would do [ … ] I was really lucky that I have clinical supervision from a consultant psychiatrist once a week, [ … ] So having that is key I think to feeling comfortable in the role, and also growing those diagnostic skills as well, which have never really been put to the test before” [P05, CMHT]*

#### Approaching sensitive topics

Four participants (n=4/20), including two with management roles alongside being prescribers described in their view how some pharmacists may not feel comfortable asking difficult or sensitive questions such as about sexual dysfunction, self-harm and suicide, and sedation. For example, they described how some may hesitate to ask about sedation as a side effect to medication unless they saw visible signs, due to insufficient training. This was explained as possibly being due to pharmacists not being exposed to various situations where they could learn how to ask these difficult questions during their training, which made them feel uncomfortable.

Another pharmacist prescriber, who also supported other PCN pharmacists mentioned that, in general, they felt that pharmacist prescribers found it challenging to communicate with patients with mental illness and manage their mental health care, particularly when compared to physical health problems.

#### Lack of support

A lack of support either from other pharmacists working in the same role or from wider professionals working in the same team was discussed by few participants as being apparent at the beginning of their prescribing role. One pharmacist explained how this impacted negatively leading to reduced job satisfaction and professional isolation. Another pharmacist working in a perinatal mental health team explained that they felt isolated as they were employed outside the pharmacy team and there were no other pharmacists in their team to interact with for support.

*“So I think working in a community team I’m not employed by the pharmacy department, I don’t sit in the pharmacy department, I’m completely separate and part of the MDT [multi-disciplinary team] for perinatal. So I end up interacting with other professionals that don’t necessarily see things from the same perspective or aren’t working in the same way so it’s a little bit more challenging to soundboard and get support.” [P09, perinatal CMHT]*

However, despite this challenge this particular participant went further and explained this issue also enabled them the opportunity to build a good network with other professionals such as medical prescribers.

### Training and education

Training related issues were discussed by the majority of the respondents (n=17/20). These were categorised as training relating either to their non-medical prescribing course or their undergraduate pharmacy programme, or both.

#### Non-medical prescribing training course

Several issues related to the content of participants’ non-medical prescribing training programmes were reported.

A general practice pharmacist who had a second role in a CMHT reported not actively prescribing for patients with mental health illness straightaway after qualifying as a non-medical prescriber, as they felt their training course did not adequately prepare them for the role and build competence in mental health, or to teach them how to assess patients with mental health issues.

*“So, once you get your prescribing qualification the six month course that you get, doesn’t actually equip you with the ability to prescribe, it gives you the tools to do so. I still felt like you need to … I needed to build competency in the particular area and have experience with dealing with those patients under a guide of a GP before I was let loose on prescribing. So, that’s why it took about six to seven months to get that sort of experience [ … ] So, another thing is that the course itself doesn’t actually help you in a particular clinical area, so if your clinical area is mental health, they don’t really give you any specific guidance on assessing a patient for mental health.” [P13, general practice]*

While participants expressed a willingness to improve their competencies in mental health care, they described either that their training did not fully support their development in such an area, or there was uncertainty in how to expand their competencies in mental health.

*“It’s quite unclear how we’re actually meant to expand our competencies. It’s, sort of, just when we feel confident almost, we can say, we’re going to prescribe in this area. But I think for mental health, with it being quite a specialist area, for me to actually put my name on a prescription, I would want to have had some really thorough training. And it’s not always clear how to access that, or what training that should be.” [P18, general practice]*

A CMHT pharmacist noted that their non-medical prescribing training course lacked coverage of essential clinical aspects, such as risk assessment and mental state examinations. To address this gap in knowledge and skills, a minority of participants commented that practical shadowing was invaluable in providing these assessment skills.

A few pharmacists believed that the non-medical prescribing training course was good enough for them to be a safe prescriber, and that other more practical aspects should be led by the prescriber themselves through experience and learning.

*“I think that the course is what you make it really. So it depends on what your DP [designated practitioner] input is like and the teams that you work in, but it also depends on how proactive you are. It’s pretty much led by yourself, so you have to be actively seeking out experience, and it is quite a lot of onus on the person to develop their role and areas of competency. So yes, the course is good, it gives you a good framework, but the filling out of that framework has to be led by you. So I think that the efficacy of the course is user-dependent really.” [P14, CMHT]*

A general practice pharmacist argued that clinical aspects some participants felt were lacking in the non-medical prescribing training course were already addressed in undergraduate education. This in their view justified why the non-medical prescribing course could focus on other areas, such as communication skills, rather than clinical skills. Another participant mentioned having a good mentor in mental health to shadow was helpful since the non-medical prescribing training course did not cover mental health conditions as thoroughly as physical health conditions.

#### Undergraduate pharmacy programme

Reported challenges associated with the undergraduate pharmacy training programmes were that they were considered theory-driven and not focussed on practical training, which resulted in pharmacists not having adequate exposure to practice in different pharmacy sectors. These issues were discussed by pharmacist prescribers and those who had joint roles as managers, and was reported to lead to a lack of ability in dealing with patients effectively in real-life situations.

*“Talking about the undergraduate course, we definitely don’t have enough placements et cetera. [ … ], it’s very theory driven [ … ] you could say that you would probably need some more time actually in the real life scenarios. You know, that patient coming in, you don’t know what they’re going to say to you. That seems to be some of the fear as well when you’re doing, kind of, prescribing.” [P02, general practice]*

Another pharmacist based in a crisis and psychiatric liaison team went further and discussed the difference between training for doctors and pharmacy students at undergraduate level, and the relative lack of exposure to practice settings in pharmacy which included with patients and how to manage their needs. This was compared to medical students who they said were frequently based on wards and rotations where they were expected to be dealing with patient related problems, resulting in newly qualified doctors practising more confidently compared to pharmacists.

*“It’s not the same when you compare a junior doctor that has been constantly in the wards, in the hospital, seeing patients, having to manage them and, on top of that, doing their training. So, you’ve got pharmacists that are very chemistry based and pharmacology based but with a small contact with patients or their management [ … ] Theory is very different from practice.” [P06, crisis and psychiatric liaison team]*

A participant also commented on the potential challenges associated with preparing UK pharmacy students to be ‘prescriber ready’ at graduation starting in 2026, and discussed the uncertainty in how prescribing training would be integrated into the new MPharm curriculum and whether new graduates would be sufficiently prepared and confident to prescribe safely in practice.

### Ambiguity

#### Role clarity

Over half of the participants reported either a lack of understanding or misunderstanding of what pharmacists can do from both other staff and patients as a barrier to delivering non-medical prescribing services. One general practice pharmacist mentioned that both GPs and patients were not aware of the pharmacists’ own expertise in mental health, which adversely affected their clinic’s development and progress. Some reported that doctors tended to refer patients with non-pharmacological needs to them as they underestimated what skills pharmacists have.

*“One of the barriers could be that getting GPs, so doctors, to understand the value of a pharmacist with expert knowledge in mental health and a prescribing qualification [ … ] I think that’s been a big challenge [ … ] It may just be the fact that they do not have the awareness what a pharmacist could do with the specialist in mental health. But this is one of the barriers I found to … for my development of my clinics. And again, the barrier of patients not understanding. So, when they … if a patient has a mental health problem, whatever, they want to see the GP that they know best, so again, it’s about managing expectation or educating patients that a pharmacist may have specialist knowledge and have the ability to address their mental health needs.” [P13, general practice]*

Additional concerns related to role clarity were raised by a small number of participants, who highlighted what they considered to be a restricted scope of practice (n=7), a lack of a well-established job structure for pharmacist prescribers to follow (n= 4), and an unclear job description (n=2). One general practice pharmacist prescriber noted that having a restricted scope of practice was not practical without clear guidance on how to expand this in the future.

Another pharmacist prescriber and manager explained that there was difficulty across different therapeutic areas, including mental health, to encourage pharmacist prescribers to prescribe within their scope of expertise and understand their boundaries.

*“… there’s been challenges in first of all getting staff to prescribe within their competencies.” [P04, PCN]*

A pharmacist working in CMHTs discussed a lack of clear job description and guidance from the team when they started a prescribing role, which alongside their own uncertainty as a new prescriber about where they best fit within the group led to the team referring what the pharmacist considered to be inappropriate patients to them to manage. As a result they also found it difficult to determine where they best ‘fitted’ into the team.

*“I think when I first went into my role, it was a little bit ill-defined, it was a very broad job description. So it was a little bit of a struggle trying to find exactly where I was fitting in with the team because they’d never had a pharmacist in post before. And I think that combined with a lack of experience using my prescribing role meant that it took quite a while for the role to sort of bed in really. And there was a bit of confusion in the team about exactly what I would be doing. So there was a bit of a misconception that it would just be the same as a medic [doctor], basically, and they were sending quite inappropriate patients for me to review at first.” [P14, CMHTs]*

A pharmacist prescriber and manager discussed that they developed a document detailing the competencies and roles of pharmacist non-medical prescribers to support staff who were not familiar with them and what they can do. This recorded prescribers’ competencies and tracked their development over time, helping other staff understand what items they could be asked to prescribe and duties to carry out. An enthusiastic and supportive multi-disciplinary team to work with was also reported by 4 participants to be helpful in developing pharmacist prescriber roles as well as enabling the team to understand what pharmacists can do. A pharmacist working in CMHT reported how working closely with the broader team encompassing doctors and other healthcare professionals was crucial in them understanding the pharmacists’ role and capabilities.

#### Guidelines and signposting

A few pharmacist prescribers discussed the perceived ambiguity they felt existed in mental health guidelines when compared to other physical health conditions. A pharmacist prescriber with mental health speciality in general practice who also had an educational role discussed how this ambiguity led to prescribers being uncertain in how to discontinue mental health medications and what the next steps should be.

*“The other thing that mental health guidelines, unlike say cardiovascular ones, is there is more fluidity in them. So, a cardiovascular guideline and an asthma guideline, the asthma guideline, great, steps, step one, do this, step two, do this, step three, do that et cetera, et cetera, step four [ … ] Whereas within mental health, if you’re looking at the depression guidance and NICE guidance, when you go into it, they don’t go [ … ] they’re going to go talking therapies, what do you mean by talking therapies, you mean talking therapies and they give some examples, oh by the way, CBT [Cognitive Behavioral Therapy] [ … ], but then they go, oh moderate to severe depression list, that and an antidepressant. Then it’s a bit vague about what to do next, do you increase the dose, do you wait, the actual steps in the process of what you would consider [ … ], a lot of the advice around how to reduce and stop psychotropic medicines is rubbish. It’s really vague, some of that is really impractical situations.” [P01, general practice]*

A pharmacist prescriber based in general practice mentioned that as a result of a lack of perceived standardised guidance for use of anti-depressant medications, patients might receive different levels of care. These different levels of care were said to stem from variations in pharmacist prescribers’ willingness and confidence in making medication changes, influenced by their experience and the lack of standardised guidance.

A lack of clarity on where to signpost patients to in crisis or which local services to refer patients to was mentioned as a challenge by only two participants. A prescriber who was also managing PCN pharmacist prescribers reported that they felt there were lots of pharmacist prescribers who lacked the clarity in where to signpost patients who experienced mental health crisis. Another pharmacist working in a CMHT highlighted the importance of having adequate knowledge about available services to effectively signpost and refer patients to for further support and advice when needed for any purpose.

### Workload management

#### Operational and resource issues

Issues related to staffing/resources, administration, and diary management were discussed by some of the pharmacist prescribers.

Seven participants discussed staffing and resource limitations as they reported that there were not always adequate numbers of prescribing pharmacists available. A CMHT pharmacist raised concerns regarding being the only pharmacist available which they felt was not enough given the number of patients that needed to be seen across the area they worked in. Consequently, the shortage of pharmacist prescribers meant there was nobody to cover their absence if on leave, which led to concern about what may happen to service provision in this situation.

*“where I work, [ … ] one of the largest (counties) in the country. [ … ] I am the only pharmacist working within that pathway. Even in our smallest teams, there is probably a caseload of about 300 people at any one time, and that can go up and down. [ … ] So if I take annual leave, there is nobody who covers me when I’m on leave. If I’m off sick, there’s nobody who covers me when I’m off sick. So it felt like setting something up with that in mind would have fallen apart quite quickly.” [P17, CMHTs]*

A CMHT pharmacist believed that because there was a smaller number of pharmacists compared to medical and nursing staff, they were not recognised for the work they were doing as a prescriber. Other pharmacists discussed the importance of pharmacist prescribers being better recognised and remunerated, noting a perceived gap despite their training and prescribing responsibilities. However, a general practice pharmacist argued that pharmacists should not be paid equivalently to doctors, due to their different undergraduate training, with suggestions that this salary gap reflects the differences in their experience and skills.

A little more than a quarter of pharmacist prescribers expressed concerns related to administrative issues, including identifying high-risk patients to target, managing appointments, and typing letters. An perinatal community mental health team pharmacist highlighted the lack of administrative support for pharmacists, unlike doctors who received such support, as a key challenge in service provision. This lack of administrative support resulted in increasing their workload and working hours.

*“One of the difficulties often is admin[istrative] time and admin help, so, often that’s not factored in. When they get new doctors, they’ll often factor in that they need extra admin perhaps to type letters, send appointment letters, that sort of thing, and I have found that with me often that wasn’t considered.” [P07, perinatal CMHTs]*

Issues relating to the management of work diaries was reported among a similar number of participants. A CMHT pharmacist mentioned that due to what they considered to be the lengthier time taken for each mental health review compared to physical health reviews (which they said was due to extra time spend discussing emotions/feelings), this created challenges with diary management limiting the number of patients they could see per week.

*“Diary management is a massive challenge as well because I don’t actually see that many people in a week because when I review someone, say the preparation might take an hour, the review itself might take an hour and it might take me an hour or more to do the write up, the letter to the GP and to liaise with any other sort of services or professionals involved with that patient.” [P05, CMHTs]*

Another pharmacist working in a perinatal CMHT explained that the increased workload arising from lengthy patient reviews resulted in them having to reduce the number of appointments per day to cope with this challenge, which extended the waiting time for patients to be seen.

#### Service impact

Out of 20 participants, only one reported that there was a formal evaluation of the services they provided for people with mental illness. This evaluation involved analysing service data such as patient referrals, treatment outcomes, and clinical notes, which was shared with trust management to support a new role and disseminated via a journal article). A further three participants referenced the use of patient surveys to explore the impact of their service on care (though no official evaluation was conducted). Despite noting a lack of formal assessment, the majority of participants shared insights into the perceived impact of their service.

Participants reported that their patients’ feedback indicated that the pharmacist was highly valued and appreciated. They included examples of how the pharmacist spent more time with patients to discuss their problems, allowing patients to be involved more in the decision-making process, and offered a more holistic review. Respondents also discussed how their patients were reported to say how they talked in lay language and in a friendly and patient way, which made it easier to follow and understand.

One pharmacist based in general practice justified offering more time (from 20 to 30 mins) to patients compared to other professions by explaining that, unlike GPs who they thought were restricted to 10 minute appointments for the entire consultation, pharmacists had more capacity with their appointment to provide thorough care. [Perceived impact.]

Various positive outcomes of their prescribing in various settings were described by more than half of the participants (n=11), including frequent de-prescribing of medicines which resulted in successful reduction, and in some cases, stopping inappropriate medication. This was reported to lead to patients experiencing fewer side effects and reduced their medication burden as a result. [Perceived impact.]

Providing education to staff working with pharmacist prescribers, and reducing doctor’s workload to focus more on complex cases were the main benefits for other health professionals reported by participants that arose from introducing pharmacist prescribing services. [Perceived and documented impact.]

However, a CMHT pharmacist prescriber with a joint role as an education and training lead argued whether them providing care helped in reducing the GPs’ workload due to them still needing to refer patients to their service. [Perceived impact.]

*“If the drugs aren’t working, the patient keeps going back to the GP. The GP has to see the patient lots and lots because the drugs haven’t worked. They still have to refer them into us. The referral pathways are still hideous for patients, they’re still very slow. No, I can’t imagine it is saving workload for the GPs.” [P03, CMHTs]*

## Discussion

### Key findings

This study explored the impact of, and factors influencing, the implementation and delivery of pharmacist non-medical prescribing services for patients with mental illness in community-based settings in the UK, an area where, to our knowledge, few studies have been conducted. A total of 20 pharmacist prescribers, each with different roles across various care settings, discussed several factors that influenced the successful implementation and delivery of the services they provided. Four main themes emerged from the discussions with the participants concerning factors influencing service implementation and delivery: insecurity, training/education, ambiguity, and workload management. Under each main theme, various subthemes were identified. These included challenges with confidence in prescribing (under insecurity), insufficient preparation for supporting people with mental illness in their training (under training/education), and a lack of clarity regarding pharmacist roles and expertise from patients, other healthcare professionals, and relevant guidelines/other documents (under ambiguity). When considering the impact of their care, most of the participants included in the study reported that they were unaware of any formal evaluation of the services they provided but did discuss a number of perceived benefits they felt their services created.

The role of pharmacists is evolving to include greater involvement in multidisciplinary teams (MDTs), reducing polypharmacy and inappropriate prescribing, and performing medication reviews (21, 28, 29). While published evidence highlights this broader role, much of it either does not focus on mental health or is outdated (e.g., from 2014). Although the literature acknowledges the expanding role of pharmacists, there is limited research exploring the factors that influence pharmacist prescribers who care for patients with mental illness. To our knowledge, previous research exploring non-medical prescribing by pharmacists for people with mental illness has focussed on isolated drugs, for example the factors that influence pharmacists when reviewing Z-drug hypnotics (30). In contrast, this research provides more broadly the factors that influence the care pharmacist prescribers provide in community settings when managing patients with mental illness. This understanding of the influencing factors has the potential to assist and guide policymakers with workforce planning, in particular in driving the integration of community based health services including PCNs and Integrated Care Boards (ICBs) in the UK National Health Service. Addressing the identifed challenges around role clarity, empowerment and education/training may help support and optimise the expanding role of pharmacists. This aligns with the NHS Long Term Plan’s ambitions, which highlights the increased need for pharmacists in providing integrated, person-centred care, especially in mental health services (31).

A recurring subtheme under insecurity was the lack of confidence in prescribing abilities, noted by most participants, whether at the beginning of their roles or as an ongoing challenge. This mainly stemmed from participants feeling not fully competent and lacking the required knowledge and skills to prescribe for people with mental illness. This is a significant concern as confidence in prescribing is important for ensuring effective, safe and rational use of medicines. This finding of low confidence in prescribing is supported by a scoping review that explored 33 studies examining prescribing competence and confidence across pharmacy and medical disciplines (32). The review found that even when pharmacists felt competent, they often still lacked confidence in their prescribing abilities. However, only 9 of the included studies focussed specifically on pharmacists, and even fewer explicitly highlighted a lack of confidence in prescribing. Notably, the majority of these studies were not conducted in mental health settings but focussed on broader or general populations, highlighting a gap in the literature around pharmacist confidence specifically in mental health prescribing contexts. This issue is not unique to pharmacy; alongside pharmacists, nurses prescribing in primary care across the UK, as discussed in Edwards et al. (33), raised similar challenges related to a lack of self-confidence when they first began prescribing. Addressing this gap is important, as greater confidence in prescribing may lead to improved patient care, particularly in mental health, where safe and effective medication management is critical.

Having sufficient experience as pharmacists was highlighted as essential in a study involving junior (pre-registration) pharmacists across the UK, where participants reported that confidence could be developed through increased clinical experience, which they viewed as necessary before prescribing confidently (34). Similar findings emerged in our study, where participants emphasised the need for more experience to build prescribing confidence. Fisher et al. (35) also explored this theme in a study conducted across all 14 NHS health boards in Scotland, using focus groups and semi-structured interviews. Their findings revealed that while competence was perceived as important for confidence, it was experience that played a more critical role. Although hospital pharmacists felt competent to prescribe, their confidence only developed with time and practice. While some studies have found little agreement on which factor (confidence, competence, and experience) most influences prescribing (32, 35), participants in this study described these factors as closely interconnected. This highlights the importance of gaining experience across different sectors, including both primary and secondary care, as essential for developing the confidence needed to manage more complex cases, such as patients with mental illness, as emphasised by our participants. This was also suggested in another study involving pharmacy students across the UK, which emphasised the need for more experiential learning and exposure to mental health patients to improve both competence and confidence (36).

Nearly all pharmacists in this study highlighted that inadequate training, either in their undergraduate pharmacy programmes and/or in non-medical prescriber training courses, had left them unprepared to qualify as prescribers. Key areas that were felt to be either not well covered or lacking included diagnostic skills, assessment of mental health status, and risk assessment. This gap in training and education is critical, given pharmacists’ expanding prescribing role in mental health teams, where confident, competent decision-making is vital for safe care for patients. These findings were in accordance with a systematic review published in 2020, which included 14 studies exploring the opinions and views of pharmacists on non-medical prescribing in primary care at a global level (37). Pharmacists in these studies expressed concerns about both the training that prepared them to become prescribers and the additional training available afterward, describing both as inadequate. They felt that areas such as diagnostic and consultation skills were unsatisfactory, which, in some cases, were identified as challenges to prescribing. Similar findings were found in a recent scoping review that included 64 studies examining barriers to pharmacist prescribing in the UK, Canada, and Australia, in both primary and secondary settings (38). Another scoping review of 63 studies that aimed to explore the global evidence for non-medical prescribing services (both nurses and pharmacists) for patients with mental illness, identified the same issue (20). These findings align with what we discovered in the present study, further highlighting the significant challenges pharmacists face due to insufficient training (38). This finding suggests that while non-medical prescribing courses cover legal and basic knowledge, they may not fully prepare pharmacists to prescribe independently, especially in mental health care. Participants felt underprepared for managing complex cases involving suicide risk and risk management, areas often underrepresented in their training. Similar suggestions were raised in recent studies, particularly among primary care pharmacists, highlighting the need for advanced training in clinical knowledge and diagnostic skills, with depression identified as a key priority (39, 40).

This training gap is not limited to the independent prescribing course; participants also felt that undergraduate pharmacy education lacked the practical preparation provided in medical programmes, particularly due to limited clinical placements. A 2014 study comparing pharmacy and medical curricula found that while pharmacy students outperformed their medical counterparts in pharmacotherapy knowledge, medical students demonstrated stronger practical prescribing skills (41). Although undergraduate pharmacy degree programmes in the UK were instructed in 2021/22 to better integrate prescribing training (including 90 hours of supervised practice) (10), a study exploring the views of pharmacy students across the UK in 2021 found that they felt underequipped in terms of mental health training (36). With all future pharmacy graduates qualifying as prescribers from 2026, concerns remain about whether current undergraduate training will sufficiently prepare them to prescribe confidently from registration.

Participants in this study perceived a lack of recognition and understanding of pharmacists’ roles by both healthcare professionals and patients. Medical staff were reported to undervalue pharmacists’ expertise, which may hinder integration into care teams and limit their contribution to improving patient outcomes. This echoes findings from a UK GPhC survey, where pharmacists across Britain expressed concerns about inadequate support from doctors in their roles as prescribers, which was reported to hinder their professional development (42). Similar concerns were identified in a 2021 systematic review including 14 studies which aimed to explore the perspectives of pharmacists and pharmacy graduates on non-medical prescribing, suggesting that some doctors’ reluctance to accept pharmacists as prescribers may stem from viewing them as a challenge to their authority and professional hierarchy (37). Peer and medical support were seen as key enablers in this study, with participants highlighting the need for more pharmacists to adopt prescribing roles to raise visibility, a recommendation also highlighted in Anderson et al.’s study (43) that explored the role of pharmacists in general practice. The author further recommended that pharmacists take broader roles, including educating stakeholders to foster understanding and collaboration (43). Ensuring that all healthcare staff understand the responsibilities of pharmacist prescribers, and supporting pharmacists in clearly defined roles, can help pharmacists focus on core tasks such as prescribing, reducing non-clinical duties and improving workload management and appointment efficiency as highlighted by participants in this study.

Participants in this study also noted that many patients were unaware pharmacists could prescribe, often preferring to see a doctor instead. In their study on non-medical prescribing from patients’ perspectives, Hobson et al. (44) attributed this to limited patient understanding of pharmacists’ qualifications as a prescriber. The author further explained that this issue stemmed from patients viewing pharmacists as dispensers, a perception also highlighted in another study (43, 44). However, this study found that patient trust often increased after direct experience with a prescribing pharmacist, aligning with a review of 17 studies that explored pharmacist prescribing from the perspectives of patients, pharmacists, and other healthcare professionals (45). This review found that confidence in pharmacist-led care grew following initial consultations. These studies, however, focussed on general populations rather than mental health settings. Despite high satisfaction of patients reported by pharmacist prescribers in this study, these outcomes were based solely on their perspectives, as patients were not included. The lack of formal evaluation in current settings limits a comprehensive understanding of service effectiveness.

Nearly all participants in this study offered specific suggestions to improve the services they provided to patients with mental illness. These suggestions guiding successful implementation and delivery of pharmacists independent prescribing for patients with mental illness are summarised in **Table 2**.

**Table 2:** Specific suggestions by pharmacist prescribers to improve services provided to patients with mental illness.

Themes for area for improvement		
Training and education	Exposure	Role clarity
1. Improve training for pharmacists working in community settings to enhance early intervention and management of mental health conditions, potentially reducing referrals to secondary care. 2. Include undergraduate and postgraduate training on: • Assessment skills, and mental state examination. • How to ask sensitive questions (e.g. suicide, sexual dysfunction). • Crisis prevention and management. • Managing risk. 3. Invest in local mental health training and education to develop pharmacists who can excel in these roles, rather than relying on individuals with limited experience. 4. Enhance access to mental health resources and support networks for pharmacists to consult when seeking advice. 5. Provide training for pharmacists with exposure to secondary care to develop skills and experience with diverse patient groups, which will be beneficial when working in primary care, where most services are provided. 6. Provide specific training programmes for primary care pharmacists who wish to specialise in mental health in order to embed this role in primary care.	1. Exposure to both primary and secondary care helps pharmacists understand the full range of mental health medications through varied patient experience 2. Gain experience with complex cases by working in specialised mental health services.	1. A clearer framework and more detailed job description outlining pharmacists’ roles to define responsibilities for the public, patients, and other healthcare professionals. 2. Establish a clear framework by allowing pharmacists to spend more time with those already in established roles.

### Future direction

This study highlights several priorities for future research and service development. Gaps were identified in training such as diagnostic skills, mental health assessment, and risk evaluation, exist in both non-medical prescribing courses and undergraduate pharmacy programmes. Future research should explore enhanced MPharm training models, including multi-sector practice experience, to increase pharmacists’ exposure to people with mental illness across diverse settings.

There is a need to develop care models that better integrate pharmacists into primary care and CMHT teams, raising awareness of their skills among other healthcare professionals, patients, and carers. Also, As new MPharm graduates will be prescriber-ready, research should also evaluate the impact of the updated degree on pharmacists’ skills and confidence in mental health care.

Participants highlighted the lack of formal evaluation of pharmacist non-medical prescribing services. Longitudinal and mixed-methods studies are needed to assess outcomes such as medication adherence, long-term clinical impact, and patient experiences. Involving patients and carers in evaluating healthcare services helps capture their experiences and can holistically guide improvements to deliver more effective, safe, and patient-centred care, as highlighted in a report published by The Health Foundation (46).

Finally, pharmacist prescribing may occur in the community following care transfer medication reviews or reconciliation. Although this was not discussed by participants, it represents an important gap. Given the recognised risks associated with discharge prescriptions from mental health hospitals (47) and WHO’s global priority on medication safety in transitions of care (48), future research should specifically examine pharmacist prescribing in this context and its potential to reduce medication-related safety risks.

### Strengths and limitations

This qualitative study has important strengths. First, to ensure coherence and reliability in the thematic analysis of the findings, independent analyses of the first ten selected transcripts were conducted by the research team and consensus reached on the coding framework before application to the full dataset. Another strength of this study was its national reach across the UK and inclusion of participants from diverse sectors of community practice. However, Despite aiming to recruit both pharmacist prescribers and other professional groups with direct contact (e.g., GPs, nurses, psychiatrists), only pharmacist prescribers participated. Some participants also held leadership or managerial roles, providing additional perspectives; however, the study did not capture broader macro-level insights into workforce planning and service delivery, which may limit understanding of how other key team members interpret the pharmacist prescribing service. There was also a lack of representation from community pharmacy participants which limits the generalisability of findings across all community settings, yet the absence of community pharmacists may reflect the low number of independent prescribers in that sector (49). Challenges were identified in previous research regarding the quality of data and rapport-building in remote interviews (50). However, in this study, these challenges were addressed by adhering to a semi-structured interview protocol, which ensured consistency in data collection but also allowed flexibility to explore new avenues. Additionally, the use of video conferencing technology allowed the interviewer to observe and respond effectively to participants’ non-verbal cues. Recruitment via the research team’s professional networks, snowballing, and targeted outreach helped achieve a broad geographical reach; however, this may have attracted participants with a greater interest or involvement in pharmacist prescribing. As advertisements were circulated widely by several people, the total number of individuals approached could not be determined, which may affect the transferability of the findings.

## Conclusion

This study is the first, to our knowledge, to explore the impact of and factors influencing successful implementation and delivery of care with a selected group of 20 pharmacist prescribers for individuals with mental health conditions in UK community settings. Although further research with a larger and more diverse sample may be needed to confirm these findings, our result indicates that key issues could impact both the establishment and operation of these services. A key challenge highlighted was the perceived gap in pharmacists’ undergraduate and postgraduate prescriber training and education concerning interacting with and caring for those with mental health illness. Addressing this gap is necessary to ensure pharmacists are equipped with the essential knowledge and confidence to prescribe effectively. Alongside training, it is important that key stakeholders (public, patients, other healthcare professionals and policymakers) are made aware of the skills and competencies pharmacists possess and the impact on care they can have, so that they can have defined roles that complement their strengths and facilitate their full utilisation in healthcare settings. Moreover, in future research should focus on exploring patient and carer perspectives, as their voices were absent in this study and evaluating pharmacist prescriber services from the viewpoint of those receiving care will be necessary to identify issues more holistically and to develop theory driven approaches to improve and tailor services to meet the needs of people with mental illness.

## Data Availability

The raw data supporting the conclusions of this article will be made available by the authors, without undue reservation.

## Supplementary material

Supplementary File 1 Interview guide.

Supplementary File 2 COREQ checklist.

Supplementary File 3 Themes and subthemes with illustrative quotations.
