# Supplementary material for: Evaluating pharmacist independent prescribing for patients with mental illness in community care: a qualitative study
Source: Front Psychiatry. 2025 Sep 8;16:1637132. doi: 10.3389/fpsyt.2025.1637132 (PMC12450940; doi:10.3389/fpsyt.2025.1637132)
Supplement: Supplementary File 1 — Interview guide. [file DataSheet1.docx]

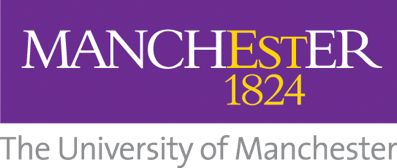


**Evaluating pharmacist independent prescribing for patients with mental illness in community care: a qualitative study**

(Interview Guide)

Date: Type of interview: (e.g., video or phone call) Interviewer:

Interviewee: Settings Position (e.g., Pharmacist or staff):

Reference No: Start time: End time:

**Part one: Introduction**

- In opening, I’d like to start by introducing myself, and extend my sincere appreciation for agreeing in taking part in this interview. The primary aim of conducting this study is to explore your experiences and perceptions of non-medical prescribing by pharmacists for patients with mental illness in the community, including the impact of this non-medical prescribing and any barriers and facilitators to delivering these services. Specifically, our objective of this interview is to identify the factors that influence the successful implementation and delivery of pharmacist independent non-medical prescribing services for patients with mental illness in the community, and to seek insight into what impact pharmacist independent non-medical prescribing has. Furthermore, it is important to be aware of that all your responses are fully anonymised to ensure your confidentiality, and I kindly request your verbal consent once more before we processed. If you have any questions before starting of the interview, please do not hesitate to ask. I will officially start the interview and begin recording. Please be mindful not to share any personal information during the interview.

**Part two: Background**

1. Could you please briefly describe your role and setting that you work in (e.g., general practice, community pharmacy, specialised mental health care). **(Are they working in primary care? Hospital? What area do they work in? how much they work? Are they part time? Do they only work a day? A week? Or do they work full time? Are they five days a week?**
2. Can you please describe any experience you have with services that involve independent pharmacist prescribing for patients with mental illness in the community? This may be experience with the implementation and/or delivery of these non-medical prescribing services, or that you work with a pharmacist prescriber in a clinical team.
3. [If a pharmacist NMP] Could you please tell me how long you have been qualified as an independent prescriber**? How long they have been a prescriber? How long they have been qualified?**
4. Could you please tell me more about the patients with mental illness that you / the service involving pharmacist independent prescribing are commonly dealing with? **Tell me about the kind of patients you look after with mental illness (what conditions they have, the types of mental illness they have? What kind of queries they ask you. What kind of help do they need**? **Mental types of mental illness you’re dealing with? what kinds of queries patients have for you? What kind of issues they are presenting with?**  **What do you do for them?**
5. Could you please tell me what sort of role they (as a pharmacist NMP) or the pharmacist NMPs they work with / manage have caring for patients with mental illness in the service?

Part three: Factors influencing the successful implementation and delivery of pharmacist non-medical prescribing for patients with mental illness in the community

1. From your experience, could you please tell me about the implementation **(early days, what is like in the early days when you were new in your role or when the service was being set up, what was it like tell me)** and/or delivery (**ongoing issues, once you have settled into the role, what was dat-to-days delivery of the service like, what did you do?, what challenges did you face kind of day-to-day just doing your job?**) of pharmacist-independent prescribing for patients with mental illness where you work? **Prompt:** Organisational, systemic barriers, if you don’t prescribe for this patient group, can you explain why? **Have you experienced any barriers or when you start you know? In your workplace (organisational or systematic barriers), You experience any difficulties when you start to when you first qualified to prescribe?- if asking the question for someone who is in direct contact with pharmacists (are you aware of any difficulties that people you work with have around starting to prescribe?- when did you start prescribing in this service? were you the first person to start prescribing as a pharmacists in this service or did they have other people before ( we want to get an idea of have they joined a service that’s already going, are they part of a new service that has been set up? Because implementation refers to the early days when the service was being set up or when they were new, whereas deliveries more about ongoing challenges, things that happen then into the future, you know, day-to-day struggles. The even after two years, three years in the role, it's kind of ongoing delivery. If he was the first one when starting the service, we might ask (what was it like kind of starting up the new service? as well as then being into a new role what was it like? How was? How did other people with you? Was it difficult? How they came to be in the role they are in now and what it has been like for them)- separate the questions about early days and then the questions for day-to-day issues.**
2. Can you describe what you think are the important factors that influence the implementation and/or deliver of pharmacist independent prescribing for this patient group where you work? Prompt: may be about training, technology, other staff members, organisational issues like finances, workload and staffing, personal factors, patient factors.
3. Could you please tell me about any impact these factors may have had? **Prompt:** Could be impact on patient care, on their workload, on working practices, on other staff?
4. Could you please tell me if there are factors that are specific to the mental health context? **Prompt:** Might be related to the medications used, the patient group, your own experience/knowledge or involvement of different services?

Part four: Impact of pharmacist non-medical prescribing services

1. If you have a pharmacist non-medical prescribing system in place where you work, do you know if it has been evaluated? If yes, can you describe this evaluation and what it found?
2. Could you please describe what, if any impact you think pharmacist non-medical prescribing for patients with mental illness has had/could have where you work? **Prompt:** Positive impact that has had on your clinical approach, patient care (Medication adherence, decision making process, accessibility, and therapeutic relationship), workload, wider organisational systems (accessibility/efficiency, and reduction in waiting time).

Part five: Recommendations

1. Could you please suggest any specific ways to improve the implementation and delivery of pharmacist independent prescribing for people with mental illness?
2. Could you please suggest any ways to further enhance the impact of pharmacist-independent prescribing for patients with mental illness?

Part five: Conclude the interview

1. Sincerely thank the participants for their valuable time in participating in this study.
2. Ask them if they would like to add any additional thoughts and comments.
3. Ask them for permission to contact them for any further queries, questions, and for participating in future studies, if interested.

**Briefly explain to participants what will happen next**: The finding of the interviews will aim to identify the barriers and facilitators in order to overcome/enhance them to optimise the

service provided by pharmacist-independent prescribers for patients with mental illness.
