# Supplementary material for: Evaluating pharmacist independent prescribing for patients with mental illness in community care: a qualitative study
Source: Front Psychiatry. 2025 Sep 8;16:1637132. doi: 10.3389/fpsyt.2025.1637132 (PMC12450940; doi:10.3389/fpsyt.2025.1637132)
Supplement: Supplementary File 3 — Themes and subthemes with illustrative quotations. [file Table1.docx]

| Theme | Subtheme | Illustrative quote |
| --- | --- | --- |
| Insecurity | Confidence | *1-“I think confidence is definitely a big one. […], fear of making a mistake. Because, for example, I worked in a hospital pharmacy, and if I ever prescribed something, then another pharmacist would check my work. Whereas community pharmacists are so rushed, and they’re not particularly clinical, so I know that once I prescribe something, it’s not going to get checked, in terms of whether it’s appropriate for that specific patient. So it’s also the element of responsibility, that I don’t feel comfortable with”* ***[P18, General Practice]***  *2- “A lot of my reviews are done over the telephone, we don’t see many patients face-to-face. […]. And that’s just because of limitations. So, in this surgery, […], I’m just upstairs, in an office with other admin[strative] staff. So, […], not seeing patients face-to-face with them, means I’m less confident in prescribing, because I don’t quite fully know the whole picture.”* ***[P18, General Practice]***  *3-“I think after so many times of, like, just checking your plan with the consultant psychiatrist and them saying yes, I agree with what you’re doing, I would have done the same thing or yes, this is really tricky, I don’t know what to do either, over time that gave me confidence that yes, what I am doing is what another reasonable person would do […] I was really lucky that I have clinical supervision from a consultant psychiatrist once a week, [...] So having that is key I think to feeling comfortable in the role, and also growing those diagnostic skills as well, which have never really been put to the test before”* ***[P05, CMHT]*** |
|  | Approaching sensitive topics | *“The fact that how comfortable people feel asking quite difficult questions. So, for example, a part of any sort of mental health review a lot of the time is suicide awareness and asking questions around […] are you thinking of harming yourself, taking your own life, which people can feel quite uncomfortable with. And I think because that’s not really covered very well, I don’t think, in a lot of the training that people do, of dealing with, for example, people who are in crisis, people that are[…] thinking about suicidal idealisation, or dealing with[…] people in psychotic episodes[…]. I don’t think people find it hugely comfortable. I think it’s quite outside of comfort zone for a lot of staff.”* ***[P04, primary care network]*** |
|  | Lack of support | *“So I think working in a community team I’m not employed by the pharmacy department, I don’t sit in the pharmacy department, I’m completely separate and part of the MDT for perinatal. So I end up interacting with other professionals that don’t necessarily see things from the same perspective or aren’t working in the same way so it’s a little bit more challenging to soundboard and get support.”* ***[P09, perinatal CMHT]*** |
| Ambiguity | Role clarity | *1.“One of the barriers could be that getting GPs, so doctors, to understand the value of a pharmacist with expert knowledge in mental health and a prescribing qualification […] I think that's been a big challenge […] It may just be the fact that they do not have the awareness what a pharmacist could do with the specialist in mental health. But this is one of the barriers I found to…for my development of my clinics. And again, the barrier of patients not understanding. So, when they…if a patient has a mental health problem, whatever, they want to see the GP that they know best, so again, it's about managing expectation or educating patients that a pharmacist may have specialist knowledge and have the ability to address their mental health needs”* ***[P13, general practice]***  *2.“I think when I first went into my role, it was a little bit ill-defined, it was a very broad job description. So it was a little bit of a struggle trying to find exactly where I was fitting in with the team because they’d never had a pharmacist in post before. And I think that combined with a lack of experience using my prescribing role meant that it took quite a while for the role to sort of bed in really. And there was a bit of confusion in the team about exactly what I would be doing. So there was a bit of a misconception that it would just be the same as a medic, basically, and they were sending quite inappropriate patients for me to review at first”* ***[P14, CMHT’s]***  *3- “Scope of practice chat from the [Royal Pharmaceutical Society] and from the [General Pharmaceutical Council] are actually creating barriers to people being able to practice […] if you have got a defined scope of practice, how do you then expand that?”* ***[P02, general practice]***  *4- “We don’t have anything kind of structured around, this is what it [prescribing role] should look like. But we don’t have any unique restrictions on what we do”* ***[P17, CMHT’s]*** |
|  | Guidelines and signposting | *1-“The other thing that mental health guidelines, unlike say cardiovascular ones, is there is more fluidity in them. So, a cardiovascular guideline and an asthma guideline, the asthma guideline, great, steps, step one, do this, step two, do this, step three, do that et cetera, et cetera, step four […] Whereas within mental health, if you’re looking at the depression guidance and NICE guidance, when you go into it, they don’t go […] they’re going to go talking therapies, what do you mean by talking therapies, you mean talking therapies and they give some examples, oh by the way, CBT[…], but then they go, oh moderate to severe depression list, that and an antidepression. Then it’s a bit vague about what to do next, do you increase the dose, do you wait, the actual steps in the process of what you would consider […], a lot of the advice around how to reduce and stop psychotropic medicines is rubbish. It’s really vague, some of that is really impractical situations”* ***[P01, general practice]***  *2-* *I think knowing the landscape of mental health services helps as well. So, they don’t feel like this is all on them, they understand how they can share a burden or refer on, or manage crisis as well. Because that’s the other thing, a lot of people don’t know how to manage mental health crisis compared to a physical crisis, where it would be ring 999, get the de-fib out. Someone talking about killing themselves and actively, sort of, thoughts around that, people find that a lot more difficult to deal with and understand. And it’s perception around what they’d do in that situation.”* ***[P04, primary care network]*** |
| Training and education | Non-medical prescribing training course | *1-“So, once you get your prescribing qualification the six month course that you get, doesn't actually equip you with the ability to prescribe, it gives you the tools to do so. I still felt like you need to…I needed to build competency in the particular area and have experience with dealing with those patients under a guide of a GP before I was let loose on prescribing. So, that's why it took about six to seven months to get that sort of experience[…]* *So, another thing is that the course itself doesn't actually help you in a particular clinical area, so if your clinical area is mental health, they don't really give you any specific guidance on assessing a patient for mental health”* ***[P13, general practice]***  *2- I think that the course is what you make it really. So it depends on what your DP [designated practitioner] input is like and the teams that you work in, but it also depends on how proactive you are. It’s pretty much led by yourself, so you have to be actively seeking out experience, and it is quite a lot of onus on the person to develop their role and areas of competency. So yes, the course is good, it gives you a good framework, but the filling out of that framework has to be led by you. So I think that the efficacy of the course is user-dependent really”* ***[P14, CMHT]*** |
|  | Under-graduate pharmacy programme | *1.If you're talking about the undergraduate course, we definitely don’t have enough placements et cetera. Do you know, it’s very theory driven […] you could say that you would probably need some more time actually in the real life scenarios. You know, that patient coming in, you don’t know what they're going to say to you. That seems to be some of the fear as well when you're doing, kind of, prescribing”* ***[P02, general practice]***  *2.“it’s not the same when you compare a junior doctor that has been constantly in the wards, in the hospital, seeing patients, having to manage them and, on top of that, doing their training. So, you’ve got pharmacists that are very chemistry based and pharmacology based but with a small contact with patients or their management […] Theory is very different from practice”* ***[P06, crisis and psychiatric liaison team]*** |
| Workload management | Operational and resource issues (staffing/ resources, administration, and diary management | *1-“The last challenge that is worthy of mentioning is the resource that I have within my pathway. So the geography of where I work, I work in the county of Northumberland, which is one of the largest in the country. […] I am the only pharmacist working within that pathway. Even in our smallest teams, there is probably a caseload of about 300 people at any one time, and that can go up and down. […] So if I take annual leave, there is nobody who covers me when I’m on leave. If I’m off sick, there’s nobody who covers me when I’m off sick. So it felt like setting something up with that in mind would have fallen apart quite quickly.”* ***[P17, CMHTs]***  *2-“One of the difficulties often is admin[istrative] time and admin help, so, often that's not factored in. When they get new doctors, they'll often factor in that they need extra admin perhaps to type letters, send appointment letters, that sort of thing, and I have found that with me often that wasn't considered.”* ***[P07, perinatal CMHT’s]***  *3-“Diary management is a massive challenge as well because I don't actually see that many people in a week because when I review someone, say the preparation might take an hour, the review itself might take an hour and it might take me an hour or more to do the write up, the letter to the GP and to liaise with any other sort of services or professionals involved with that patient” ”* ***[P05, CMHTs]*** |
